# On the Use and Construction of Wi-Fi Fingerprint Databases for Large-Scale Multi-Building and Multi-Floor Indoor Localization: A Case Study of the UJIIndoorLoc Database

**DOI:** 10.3390/s24123827

**Published:** 2024-06-13

**Authors:** Sihao Li, Zhe Tang, Kyeong Soo Kim, Jeremy S. Smith

**Affiliations:** 1 School of Advanced Technology, Xi’an Jiaotong-Liverpool University (XJTLU), Suzhou 215123, China; sihao.li19@student.xjtlu.edu.cn (S.L.); zhe.tang15@student.xjtlu.edu.cn (Z.T.); 2 Department of Electrical Engineering and Electronics, University of Liverpool, Liverpool L69 3GJ, UK; j.s.smith@liverpool.ac.uk

**Keywords:** indoor localization, Wi-Fi fingerprint database, UJIIndoorLoc

## Abstract

Large-scale multi-building and multi-floor indoor localization has recently been the focus of intense research in indoor localization based on Wi-Fi fingerprinting. Although significant progress has been made in developing indoor localization algorithms, few studies are dedicated to the critical issues of using existing and constructing new Wi-Fi fingerprint databases, especially for large-scale multi-building and multi-floor indoor localization. In this paper, we first identify the challenges in using and constructing Wi-Fi fingerprint databases for large-scale multi-building and multi-floor indoor localization and then provide our recommendations for those challenges based on a case study of the UJIIndoorLoc database, which is the most popular publicly available Wi-Fi fingerprint multi-building and multi-floor database. Through the case study, we investigate its statistical characteristics with a focus on the three aspects of (1) the properties of detected wireless access points, (2) the number, distribution and quality of labels, and (3) the composition of the database records. We then identify potential issues and ways to address them using the UJIIndoorLoc database. Based on the results from the case study, we not only provide valuable insights on the use of existing databases but also give important directions for the design and construction of new databases for large-scale multi-building and multi-floor indoor localization in the future.

## 1. Introduction

The rapid development of technologies for wireless communication and mobile devices has brought about a host of new applications and services, a notable example of which is location-based services (LBSs) [1]. Global navigation satellite systems (GNSSs) and cellular networks can accurately localize mobile users and devices in an outdoor environment, which, however, cannot be a viable option for indoor localization due to the lack of line-of-sight (LOS) signal paths [2]. Since the location of mobile users and devices is essential to LBS, indoor localization techniques not based on GNSS and cellular networks have been increasingly attracting attention from both researchers and practitioners.

Indoor localization can be done with and without ranging [1]. Ranging-based approaches rely on the distances between a point of interest and multiple known locations—i.e., anchor nodes like wireless access points (WAPs)—or differences of them in determining the unknown position of the point of interest through trilateration/multilateration. The time of arrival (ToA) technique uses the travel time between the unknown location of a user or a device and anchor nodes [3,4], while the time difference of arrival (TDoA) technique uses the time differences between the arrivals of the user’s signals at anchor nodes [5,6]. Instead of arrival times or their differences, the angle of arrival (AoA) technique uses the angles of signal arrivals, which can be estimated by measuring time differences of arrivals between individual elements of an antenna array [7,8]. Due to the reflections and multi-path interference introduced by indoor structures and obstructions, it is challenging to accurately estimate the arrival times or their differences of received radio signals through non-line-of-sight (NLOS) signal paths in time-based approaches. Likewise, the angle measurement in AOA techniques could be affected by indoor obstacles as well as the user’s body posture and the way of carrying devices. In large-scale multi-building and multi-floor indoor localization, the performance of ranging-based techniques cannot be comparable to that of single-floor indoor localization. Given the requirement of deploying numerous anchor nodes throughout buildings and floors, we can conclude that ranging-based techniques are unsuitable for large-scale multi-building and multi-floor indoor localization.

In ranging-free indoor localization techniques, the location of a user or a device is not estimated based on distance-related information with trilateration/multilateration. In the case of location fingerprinting, which is by far the most popular indoor localization technique, the information measured at a point of interest, e.g., received signal strength indicator (RSSI), channel state information (CSI) and geomagnetic field intensity, is used for localization, which is supposed to be unique to each location and thereby serves as a location fingerprint. Specifically, in Wi-Fi fingerprinting using RSSIs as its location fingerprints, there are offline and online phases in its operation [9]: during the offline phase, the RSSIs at known locations, called reference points (RPs) in the literature, are collected and stored in a fingerprint database; during the online phase, the current location of a user or a device is estimated based on the RPs whose RSSIs most closely match the newly measured RSSIs at the location. One of the most significant advantages of Wi-Fi RSSI fingerprinting is that it does not require additional hardware or infrastructure (e.g., costly sensors and dedicated base stations serving as anchor nodes) and, therefore, can be used in any environment equipped with Wi-Fi networks, including offices, hospitals, campuses and shopping malls, making it a cost-effective solution. For Wi-Fi fingerprinting, CSI also can be used as location fingerprints, which, unlike coarse-grained RSSIs, can provide fine-grained indicators consisting of both amplitude and phase information during signal propagation and enables even single-WAP localization. A significant drawback of CSI-based Wi-Fi fingerprinting, however, is the requirement of unique network interface cards (NICs) and device drivers for the acquisition of CSI (e.g., Intel 5300 NICs) [10].

Due to their recent evolution and penetration into many areas as new enabling technologies, artificial intelligence (AI)/machine learning (ML) algorithms are frequently used to improve the performance of location fingerprinting techniques. The K-nearest neighbors (KNN) algorithm is one of the most employed ML algorithms for indoor localization due to its simplicity and efficiency [11,12]. However, its localization performance is degraded by the complexity of indoor environments, especially in large-scale multi-building and multi-floor indoor localization, due to the increased spatial variability and dynamics of Wi-Fi signals. In contrast, deep neural networks (DNNs) can address the issues in large-scale indoor localization [13,14], which can model complex relationships between the input features and output labels and thereby efficiently handle the spatial variability and dynamic signals encountered in large-scale multi-building and multi-floor indoor localization. In addition to classical feedforward neural networks (FNNs) used in earlier works (e.g., [13]), more advanced DNNs like convolutional neural networks (CNNs) [15,16] and recurrent neural networks (RNNs) [17] are employed as well, due to their improved robustness and generalization capability.

Small-scale indoor localization covers only a single floor of multi-floor buildings or enclosed space (e.g., a classroom) and utilizes location fingerprints based on a limited number of WAPs. In such an environment, constructing and managing a fingerprint database is relatively straightforward, and most indoor localization algorithms can accurately estimate the location of a user or a device. However, it is not that straightforward to extend not only the way of constructing and managing fingerprint databases but also localization algorithms to large-scale multi-building and multi-floor indoor localization, where the characteristics and the dimension of input signals are considerably more complicated and extensive. Large-scale multi-building and multi-floor indoor localization have to address the following unique issues in comparison to their small-scale counterpart:

- Scalability: the higher dimension (i.e., the number of detected WAPs) and the large number (i.e., the number of RPs) of location fingerprints.
- Irregularity: differences in location coverage and internal structures (e.g., floor plans) among buildings and floors and uneven spatial distribution of RPs.

Note that the localization performance heavily depends on the underlying Wi-Fi fingerprint database. There are bodies of research reporting outstanding performance of their proposed indoor localization algorithms, which are based on their custom-built fingerprint databases covering limited areas with simple internal structures like corridors and Lab spaces, mainly because constructing large fingerprint databases is labor-intensive and time-consuming; even worse, most of those fingerprint databases are not publicly available. However, it is desirable to compare a newly proposed indoor localization algorithm with the existing ones on an equal basis, preferably based on publicly available, well-established fingerprint databases. For this purpose, Torres-Sospedra et al. provided the UJIIndoorLoc database [18], a large-scale publicly available multi-building and multi-floor Wi-Fi fingerprint database, which has been the most widely used fingerprint database for benchmarking multi-building and multi-floor indoor localization algorithms in the literature. Since the public release of the UJIIndoorLoc database in 2014, several single or multi-building multi-floor Wi-Fi fingerprint databases have also been released to the public, which will be discussed in detail in Section 2, but none of them have gained widespread acceptance compared to the UJIIndoorLoc database. Though there have been numerous studies on indoor localization based on the UJIIndoorLoc database, a systematic case study dedicated to large-scale multi-building and multi-floor Wi-Fi fingerprint databases has yet to be seen.

Considering the importance of the UJIIndoorLoc as a benchmark Wi-Fi fingerprint database in multi-building and multi-floor indoor localization and the vast amount of research based on this database since its public release, therefore, in this paper we take the UJIIndoorLoc database as a representative example of large-scale multi-building and multi-floor Wi-Fi fingerprint databases and carry out a case study based on it, which not only summarizes in one place all the major issues and ways to address them in using the UJIIndoorLoc database in research and practice but also provides valuable insights into the use of existing databases and important directions for future design and construction of new databases based on the comprehensive analysis of the UJIIndoorLoc database.

The major contributions of our work in this paper are three-fold:

- Through the case study, we investigate the major issues in multi-building and multi-floor indoor localization based on the comprehensive analyses of the UJIIndoorLoc Wi-Fi fingerprint database.
- Based on the results from the case study of the UJIIndoorLoc database, we discuss the issues in using the existing Wi-Fi fingerprint databases and suggest ways to address them by adopting advanced ML techniques in the context of multi-building and multi-floor indoor localization based on Wi-Fi fingerprinting.
- We also provide practical guidelines for the design and construction of new Wi-Fi fingerprint databases in the future to help both researchers and practitioners in multi-building and multi-floor indoor localization.

The rest of the paper is organized as follows: Section 2 reviews publicly available fingerprint databases that are well-known in the literature. Section 3 presents the results of the case study of the UJIIndoorLoc database. Section 4 discusses the challenges and provides recommendations in constructing and using large-scale multi-building and multi-floor Wi-Fi fingerprint databases. Section 5 concludes our work in this paper.

## 2. Review of Publicly Available Wi-Fi RSSI Fingerprint Databases

In this section we review the well known publicly available Wi-Fi RSSI fingerprint databases in the literature and provide their summary.

### 2.1. UJIIndoorLoc

UJIIndoorLoc is the first publicly available multi-building and multi-floor Wi-Fi fingerprint database, which covers a total surface of over 108,000 m^2^ of three, four or five floor buildings on the University Jaume I (UJI) campus in Castelló de la Plana, Spain [18].

The UJIIndoorLoc database provides 21,048 (The total number of records mentioned in [18] is 21,049, but the actual number of records contained in the two CSV files (i.e., “trainingData.csv” and “validationData.csv”) publicly released by the same group is 21,048.) records measured at pre-established RPs (933 in total) and random locations. To guarantee statistical independence between datasets, the validation dataset of the UJIIndoorLoc database was measured three months after the training dataset. The UJIIndoorLoc database is quite flexible in that the localization with it can be based on classification of building, floor and location identifiers (IDs), or the regression of three-dimensional (3D) location coordinates, or their hybrid given its large-scale multi-building and multi-floor nature.

Due to these advantages and its flexibility, the UJIIndoorLoc database has become the most widely used reference for benchmarking multi-building and multi-floor indoor localization algorithms in the literature (e.g., selected as the official database of the IPIN 2015 competition [19]).

### 2.2. WicLoc

The WicLoc fingerprint database covers the tenth floor of the new main building at the Beihang University in Beijing, China [20]. The floor occupies an area of about 1600 m^2^ and has 28 rooms with a size of 3.75 m × 8 m each and a circular corridor. The WicLoc database consists of the users’ daily location information, corresponding Wi-Fi RSSIs and other characteristics like users’ step counts and turns based on crowdsourcing, which could significantly reduce the labor cost for data collection. More than one hundred WAPs detected in the floor are segmented into pre-defined 2 m × 2 m grids.

### 2.3. TUT 2017 and TUT 2018

TUT 2017 and TUT 2018 are single-building and multi-floor Wi-Fi fingerprint databases from the Tampere University of Technology in Tampere, Finland, the details of which are described in [21,22], respectively. Both databases provide training and test datasets, whose record consists of RSSIs, floor, longitude and latitude. The TUT 2017 database is based on the records collected at random RPs (i.e., no grid-based or pre-established mapping) by volunteers with 21 devices in a five-floor building with a footprint of about 22,570 m^2^ (i.e., a size of about 208 m × 108 m). The TUT 2018 database, on the other hand, is based on the records collected at grid-based RPs in a three-floor building, where grid spacing of 5 m × 1 m is used for training and test datasets, respectively.

### 2.4. UTSIndoorLoc

The UTSIndoorLoc database [23] was collected in the FEIT Building at the University of Technology Sydney (UTS), which includes four basement levels. Excluding the non-public floors, the database encompasses sixteen active floors within the building, resulting in the total coverage space of about 44,000 m^2^. Out of 9494 Wi-Fi RSS samples from 589 WAPs measured at 1840 RPs, 9107 and 387 samples are allocated for training and testing, respectively.

### 2.5. SODIndoorLoc

As a supplement to the UJIIndoorLoc database, Bi et al. presented the SODIndoorLoc database, which covers three buildings with one or three floors, amounting to a total area of 8000 m^2^, and provides 23,925 Wi-Fi RSS samples from 105 single-band and dual-band WAPs measured at 1630 RPs and 272 testing points (TPs) with 21,205 samples for training and 2720 samples for testing and validation [24].

Notable improvement of the SODIndoorLoc over the UJIIndoorLoc is that it provides dense RPs the location information of pre-installed WAPs and computer-aided design (CAD) drawings of each floor and room layouts, which enables researchers to use the SODIndoorLoc database not only for classification and regression but also for clustering.

### 2.6. A Summary of Wi-Fi RSSI Fingerprint Databases

Table 1 provides a summary of the Wi-Fi RSSI fingerprint databases discussed in this section.

First, fingerprint databases can be categorized into single-floor, single-building and multi-floor, and multi-building and multi-floor databases based on their coverage.

Second, as for the way of collecting RSSIs, there are two categories of crowdsourcing, which is based on volunteers, and insourcing, which is purposefully designed and systematically carried out by the participants of the project. The former often leads to crowded RSSI records at random RPs but requires less effort since only volunteers are required. The latter may result in a more structured and tidier RSSI database, but it requires considerable labor during the construction.

## 3. A Case Study of the UJIIndoorLoc Database

**Figure 1:**
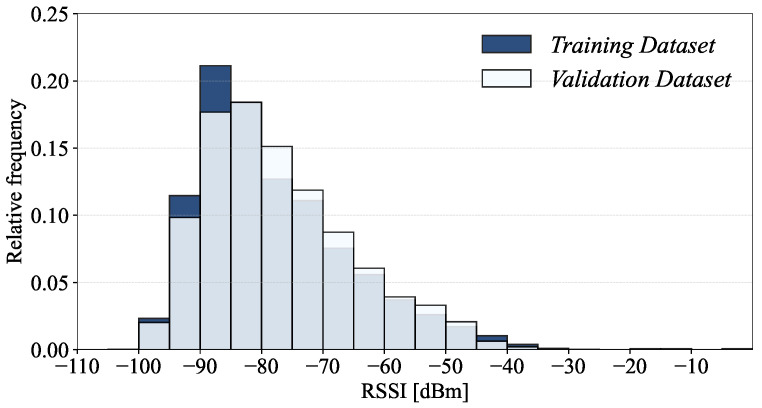
RSSI histogram of the UJIIndoorLoc dataset.

In this section, we present the results of our statistical investigation of the UJIIndoorLoc database. The UJIIndoorLoc database [18] provides 21,048 records (i.e., the collection of RSSI measurements and measurement information arranged in rows of the table) in total, i.e., 19,937 records in the training dataset and 1111 records in the validation dataset, covering the three buildings on the UJI campus, each of which consists of RSSIs from 520 WAPs and 9 fields of location and measurement information (i.e., 529 fields per record with each field representing a specific type of information arranged in a column of the table). The histograms in Figure 1 show the overall distributions of the RSSIs, i.e., in dBm [18], in the training and validation datasets.

### 3.1. Label-Based Record Analyses

The accuracy of indoor localization depends on the location and measurement information provided by a fingerprint label. Table 2 lists the fields of the UJIIndoorLoc fingerprint label, where the first six fields contain location information and the last three fields contain measurement information.

#### 3.1.1. Location Information

**Figure 2:**
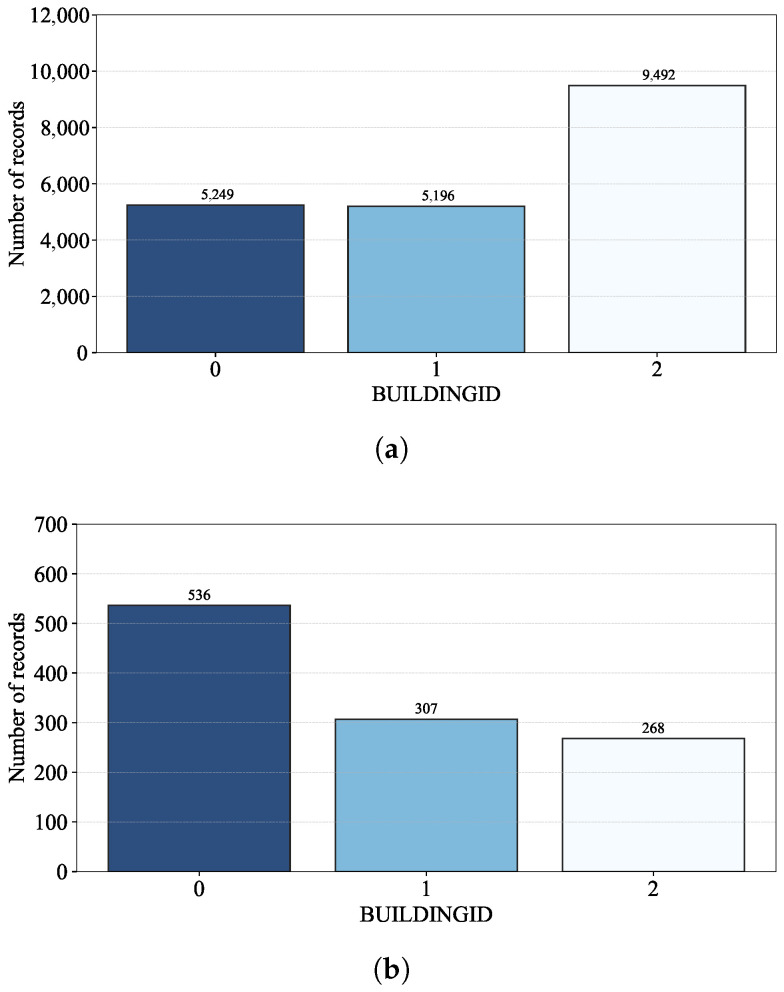
The number of records per building: (**a**) training and (**b**) validation datasets.

Figure 2 shows the number of records per building of the UJIIndoorLoc database, where there is a noticeable difference in those numbers of the training and validation datasets.

**Figure 3:**
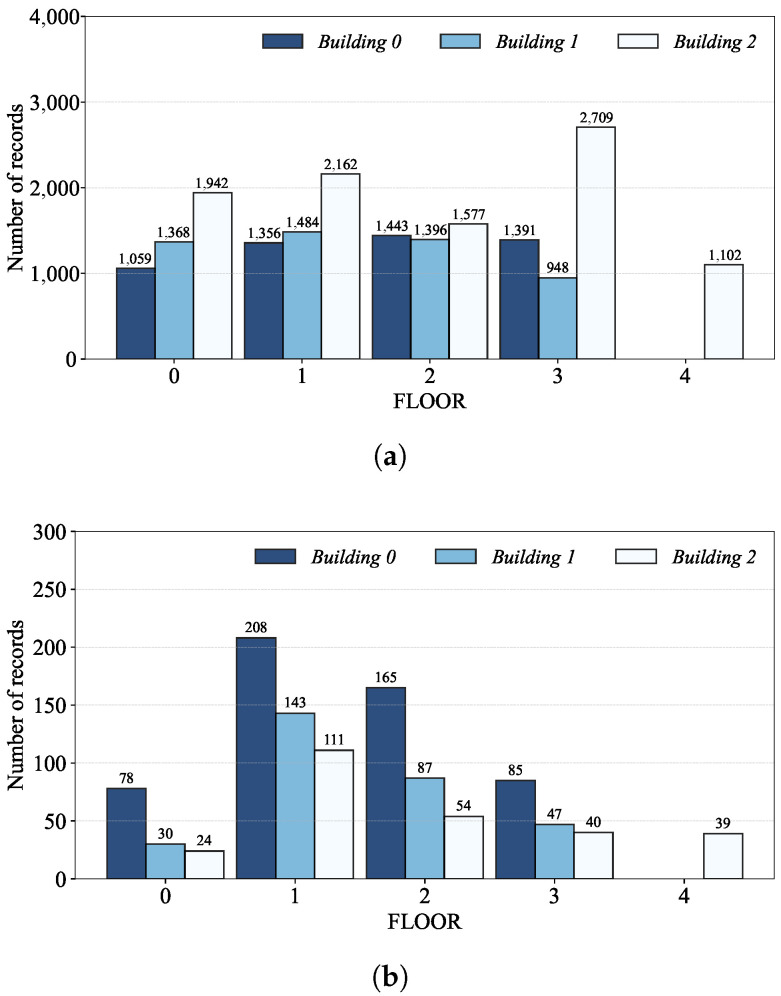
The number of records per floor: (**a**) training and (**b**) validation datasets.

Figure 2a shows that, though Building 2 have just one more floor than Building 0 and 1, its number of records in the training dataset is almost the same as the sum of those of Building 0 and 1. Regarding the validation dataset, on the other hand, it is Building 0 that has the most number of records as shown in Figure 2b. Figure 3 also shows the number of records per floor of each building; the different numbers of records per floor result from the accessibility of the rooms (e.g., the rooms on the third floor of each building are more easily accessible than the others in the training dataset).

**Figure 4:**
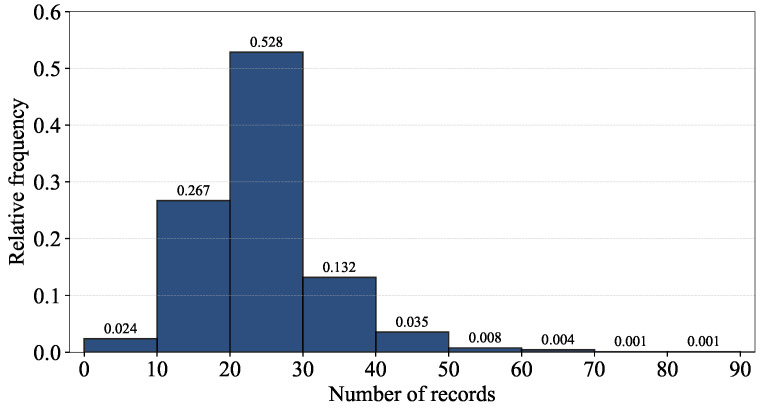
Histogram for the number of records per RP of the training dataset.

The uneven numbers of records per floor of the training dataset could be explained by its building-level RP distribution and the histogram for the number of records per RP in Table 3 and Figure 4. Note that RPs were established only for the training dataset in the UJIIndoorLoc database ([18] Table X); the records of the validation dataset do not provide SPACEID and RELATIVEPOSITION on which RPs are based. With the location coordinates of LONGITUDE and LATITUDE alone, the number of records per RP cannot be calculated.

As shown in Table 3, the number of RPs in Building 2 is significantly larger than those in Building 0 and 1, i.e., like the numbers of records shown in Figure 2a, due to the allocation of RPs for the training dataset of the UJIIndoorLoc database [18]. Considering the numbers of records per RP of the training dataset shown in Figure 4 together, we can conclude that the allocation of RPs and repeated RSSI measurements at those RPs greatly affect the numbers of records per building and floor.

**Figure 5:**
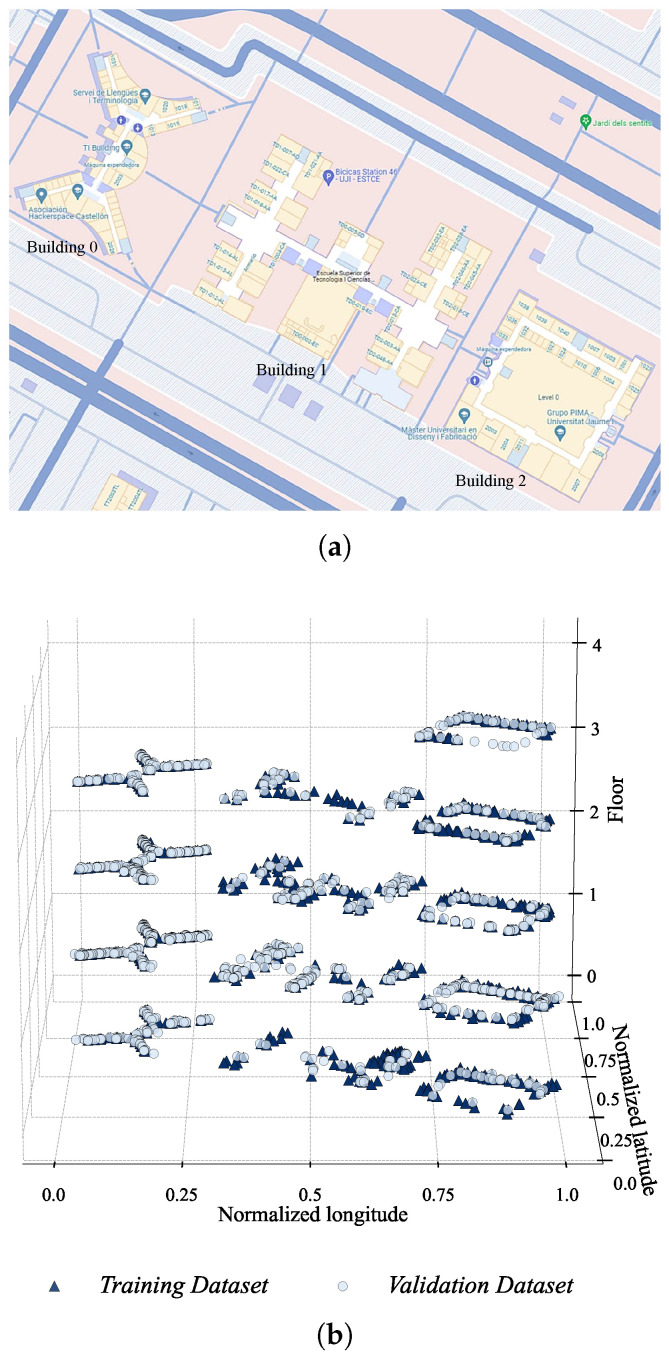
Record spatial distributions: (**a**) buildings on the UJI campus map from Google Earth, where the names of the locations are in Spanish, and (**b**) 3D visualization of the record coordinates.

Figure 5a,b show the floor plans of the three buildings on the UJI campus map with their corresponding BUILDINGIDs and the 3D visualization of record coordinates of LONGITUDE, LATITUDE and FLOOR, respectively.

Note that, though the details of capturing the real-world coordinates of RSSI samples indoors are not provided in [18], a usual practice is to convert the local coordinates of RSSI samples (e.g., *x* and *y* coordinates with respect to the corner of a building as a reference) to real-world coordinates (e.g., those based on UTM from WGS84 in the case of the UJIIndoorLoc database) based on a site plan providing the real-world coordinates of buildings.

As expected from the floor plans, the spatial distribution of the records of Building 1 is much more complicated than those of Building 0 and 2. One noticeable feature of the spatial distributions is that the coverage of the records of the training dataset is quite incomplete on the top floor of Building 2, where there are virtually no training records for the lower right part of the floor, while the validation records are more or less evenly distributed along the corridors. Such clear coverage gaps between the datasets can negatively affect the model’s localization performance on the validation dataset. Data-augmentation techniques can be used in this regard to fill the spatial gaps in the training dataset and thereby improve the generalization capabilities and localization performance of a model [25].

#### 3.1.2. Users and Phones

**Figure 6:**
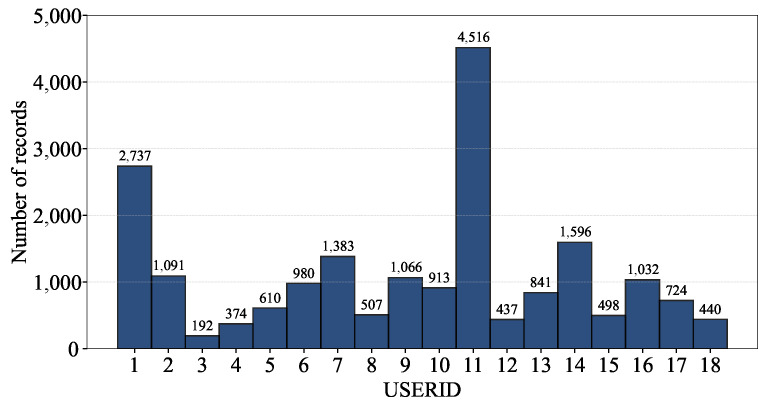
The number of records per user of the training dataset.

The UJIIndoorLoc database was constructed based on the measurements by 18 users for the training dataset (i.e., USER 1 to 18), but the USERID field was not recorded and set to 0 for the validation dataset. Figure 6 shows the number of records per user of the training dataset, where we can observe that there are a few dominant contributors like USER 11 with 4516 records and USER 1 with 2737 records.

**Figure 7:**
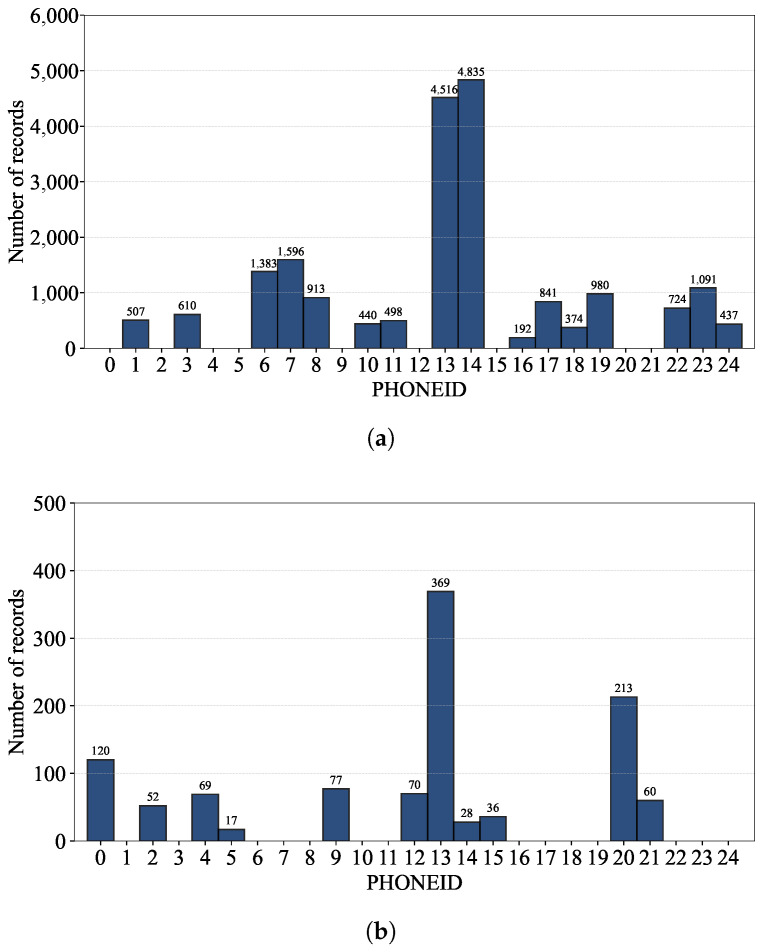
The number of records per phone of the (**a**) training and (**b**) validation datasets.

Likewise, we can also identify the dominance of a couple of phones in Figure 7, which shows the number of records per phone.

Note that the position of a user’s phone (e.g., height and direction) affects RSSI measurements as does the model and software/firmware version of the phone; at the exact same location, a combination of different user and phone could result in different RSSI values. Given the dominance of a few users and phones in constructing the database, we can also apply techniques like data augmentation [25] and data resampling [26] to reduce the risk of bias and overfitting and thereby improve a model’s generalization as discussed in Section 3.1.1.

**Figure 8:**
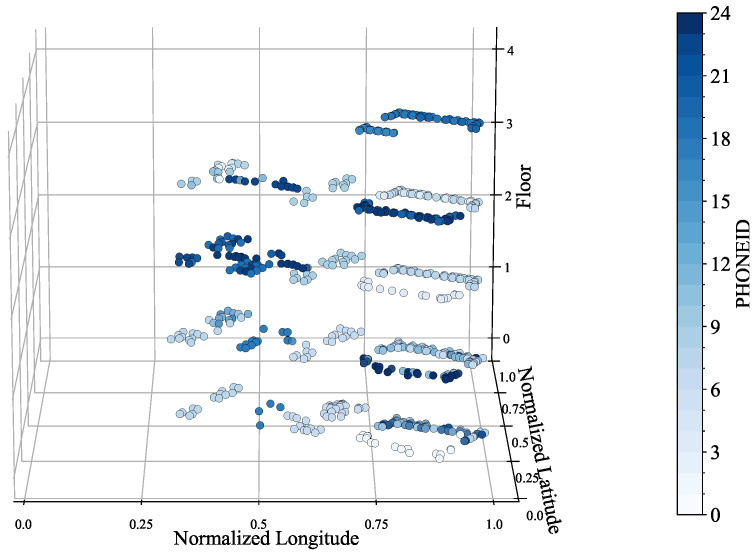
Phone spatial distribution of the training dataset (excluding PHONE 13 and 14).

The data analysis in Figure 7 reveals that PHONE 13 and 14 hold the majority of the training dataset records. Additionally, PHONE 13 is the most significant contributor to the validation dataset. Notably, there are a few instances where the same phones appear in both datasets, which challenges the model’s generalization to new, unseen phones. This observation is critical as it accurately reflects the real-world scenario, where the phones used may not be limited to those in the training dataset. Note that, without the records by PHONE 13 and 14, the remaining records of the training dataset shown in Figure 8 quite poorly cover the entire space in comparison to the whole records shown in Figure 5b.

#### 3.1.3. Timestamp

**Figure 9:**
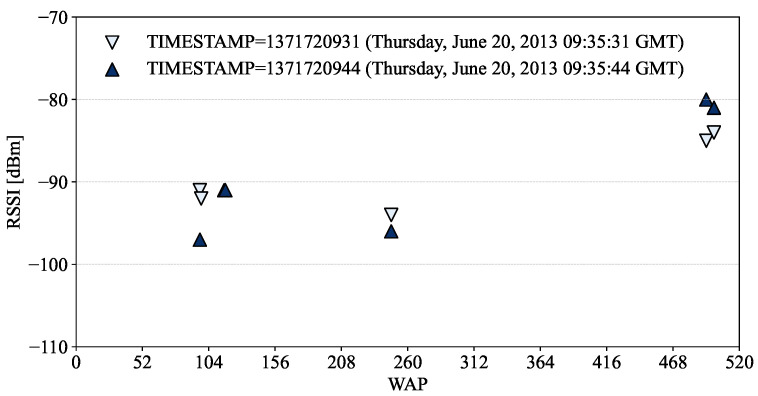
RSSIs in the training dataset measured at different times with PHONE 19 at the same location of LONGITUDE = −7300.81899 and LATITUDE = 4,864,817.599.

The time of record capture is provided by TIMESTAMP field in Unix time format, which was set by a centralized server to avoid the issue of devices’ different timing settings [18]. As shown in Figure 9, RSSIs measured with the same phone at the same location but at different times (i.e., just 13 s’ difference in the example) could be different due to several factors such as time-varying wireless channels and the movements of a user measuring RSSIs, which affects the accuracy and reliability of indoor localization systems.

This could be a major issue specifically for highly dynamic indoor environments such as airports, shopping malls and hospitals.

### 3.2. Field-Level RSSI Analyses

For the analyses of RSSIs presented in this section, we exclude the RSSI values of undetected WAPs in each record by setting them to *NaN*, which stands for “Not a Number”.

#### 3.2.1. Statistical Characteristics

**Figure 10:**
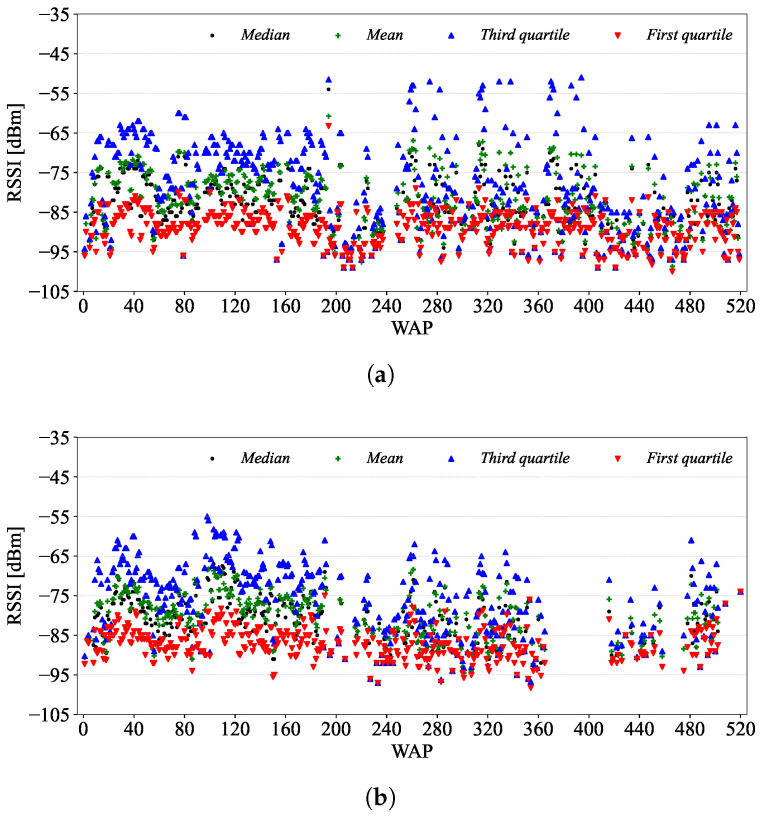
Scatter plots of the field-level RSSI mean, median and first and third quartiles of (**a**) the training and (**b**) validation datasets.

**Figure 11:**
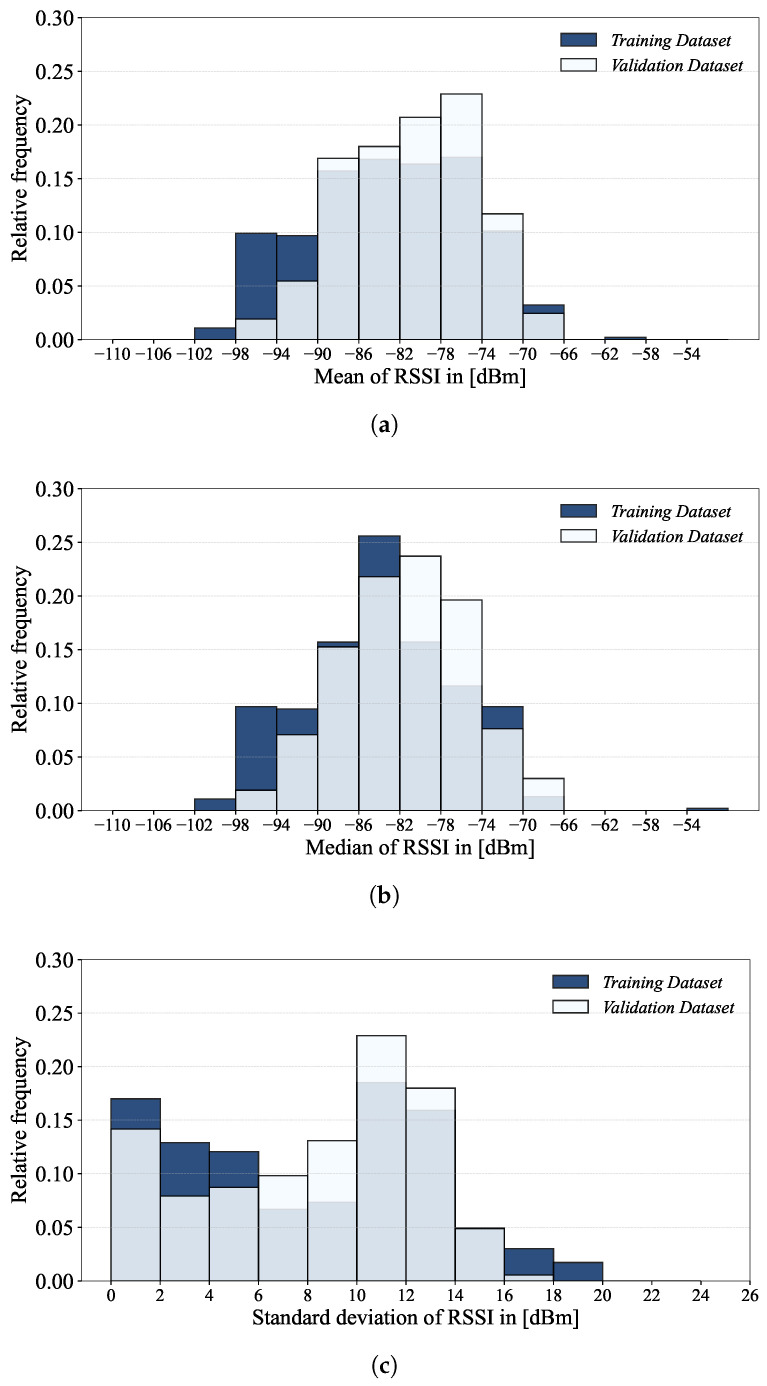
Histograms of the (**a**) mean, (**b**) median and (**c**) standard deviation of field-level RSSIs.

Figure 10 shows the scatter plots of the field-level RSSI mean, median and first and third quartiles of the training and validation datasets, while Figure 11 shows the histograms of their means, medians and standard deviations. By field-level, we mean each of the 520 RSSI columns (i.e., fields) of the UJIIndoorLoc database, the RSSIs of which are from one specific WAP.

From the field-level RSSI statistics shown in Figure 10, we can observe that most of the means and medians of both training and validation datasets fall between −95 and −70, though the spread of the training dataset is slightly greater than that of the validation dataset. As most of the RSSI values are weak (i.e., <−70) and volatile [27], they are susceptible to environmental changes such as people and furniture movement. As shown in Figure 11c, it is also worth noting that the standard deviations of the RSSIs are less than 2 for around 15% of the WAPs. This would indicate a situation where these WAPs yield similar RSSIs (e.g., they are barely detected at only a few RPs), which are of little help during the localization.

From Figure 10, we also observe that some WAPs are present in either of the datasets but not both: for example, Figure 10a shows a gap of WAPs around 240 in the training dataset, while Figure 10b shows the absence of WAPs in the range of [370, 410]. We can think of the following reasons for the inconsistencies of detected WAPs between the datasets:

- There are changes in the environments and the WAPs such as the addition of new furniture or equipment blocking the signals from WAPs and the replacement of failed WAPs with new ones. Note that a validation dataset was constructed three months after the training dataset.
- Some WAPs are not detected by certain phones due to hardware and software issues. Again, note that different sets of phones, with a few dominant ones, were used for the training and validation datasets as discussed in Section 3.1.2. The diversity of phones used in collecting fingerprints is the key to address this.
- There are significant differences in the measurement locations between the datasets as discussed with Figure 5b in Section 3.1.1.

#### 3.2.2. Unique Values

**Figure 12:**
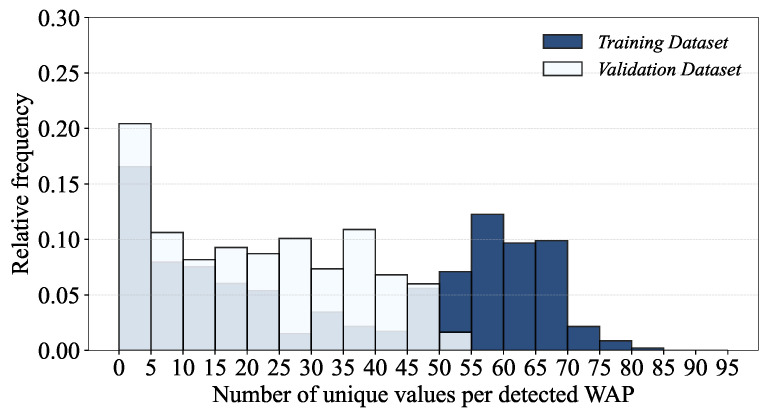
Histograms of the number of unique RSSI values per WAP.

Unique values of the RSSI from each WAP can provide an insight into the distinct characteristics of the corresponding WAP in indoor localization. Specifically, through the analyses of field-level unique values, we can identify the RSSI values that are constant over multiple locations and/or measurements (e.g., weak signals that are barely detectable) and thereby hardly contribute to location fingerprinting due to their lack of uniqueness. Those values can be removed to reduce the cost of maintaining large-scale multi-building and multi-floor Wi-Fi fingerprint databases, which not only speeds up training but also lowers the risk of overfitting. Figure 12 shows the histograms of the number of unique RSSI values per WAP.

**Figure 13:**
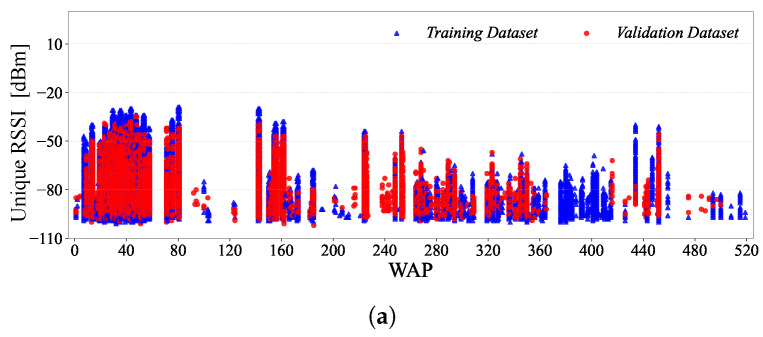

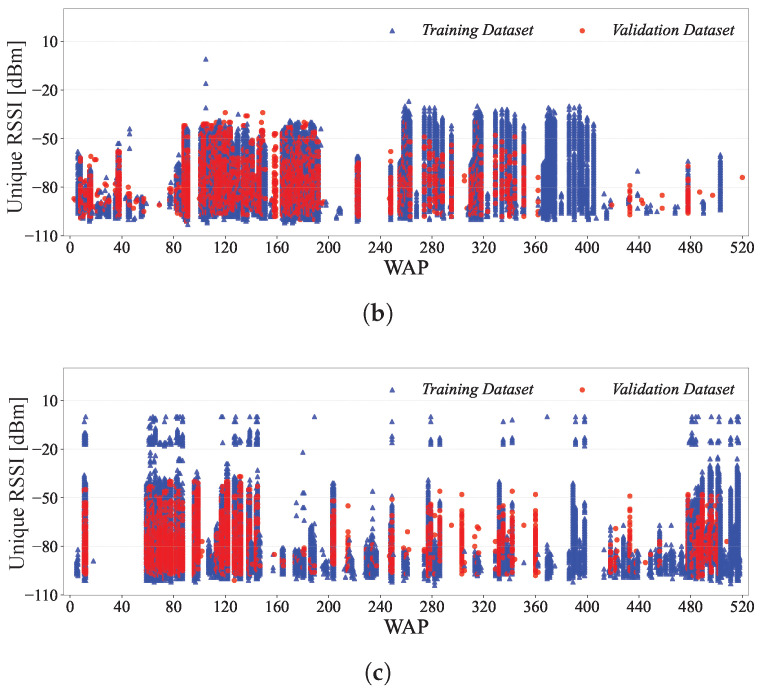
Scatter plots of unique RSSI values per WAP: (**a**) Building 0, (**b**) Building 1 and (**c**) Building 2.

The histograms reveal that a significant number of WAPs have smaller numbers of unique values; about one-third of the WAPs in the training dataset and half of the WAPs in the validation dataset have unique values of less than five. This implies that the signal strengths from those WAPs are so weak that they are detected only at a few RPs or their RSSIs are identical across many RPs. The scatter plots of building-level unique RSSI values shown in Figure 13 visualizes the significance of each WAP in a clear way.

#### 3.2.3. Spatial Distribution

**Figure 14:**
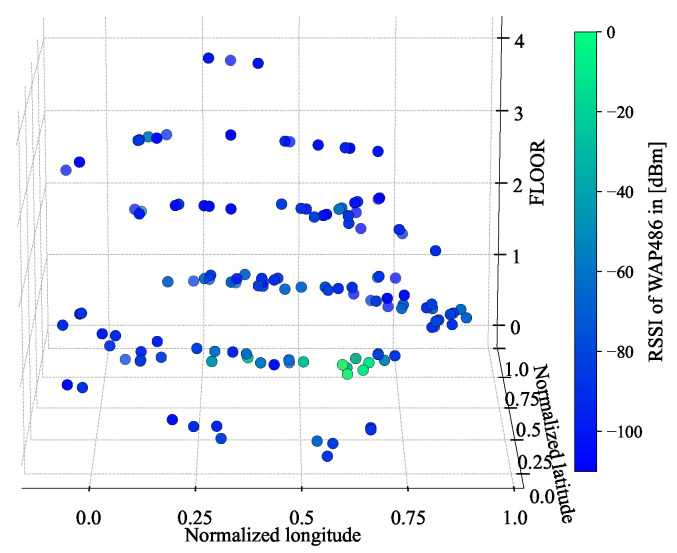
Spatial distribution of the RSSIs from WAP486 of the training dataset in Building 2.

Figure 14 shows an example of field-level RSSI spatial distributions, which is based on the RSSIs from WAP486 of the training dataset.

From Figure 14, we can observe that, though WAP486 is detected at various places, its RSSIs are much stronger on the second floor of Building 2, which would indicate its deployment location.

Estimating the locations of all WAPs in a large-scale multi-building and multi-floor environment is not practical, but our discussions based on Figure 14 provides a practical alternative: if few critical WAPs can be identified or a target area can be hierarchically divided into smaller sub-regions, we can focus on a small number of WAPs with a reduced input dimension and approximately estimate their locations through the analysis of their RSSI spatial distributions.

### 3.3. Record-Level Fingerprint Analyses

**Figure 15:**
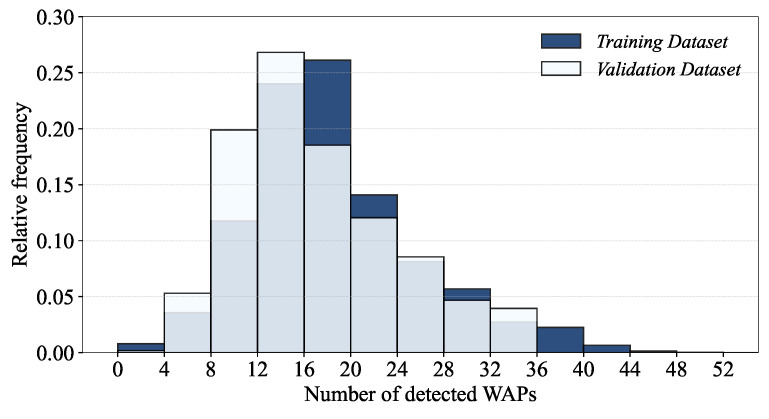
Distributions of the number of detected WAPs per record.

Due to the lack of information for SPACEID, RELATIVEPOSITION and USERID in the validation dataset, we base the record-level fingerprint analyses mainly on the training dataset. Figure 15 shows the distributions of the number of detected WAPs per record for both training and validation datasets.

The bins with the highest relative frequency for the training and validation datasets are [16, 20) and [12, 16), respectively, which is understandable given the much smaller numbers of records, users and phones and the shorter measurement period of the validation dataset.

**Figure 16:**
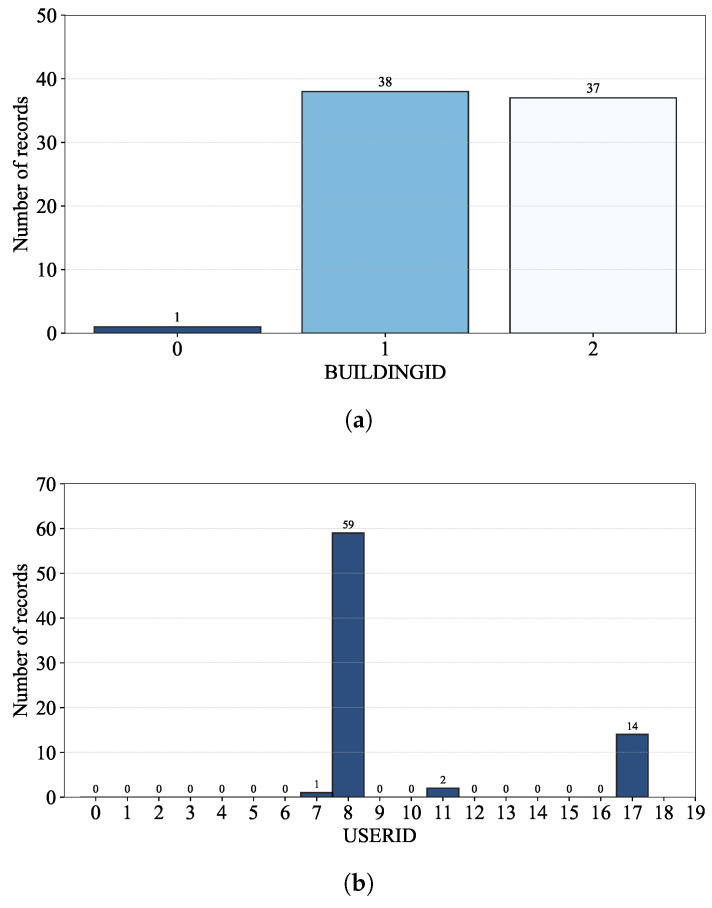

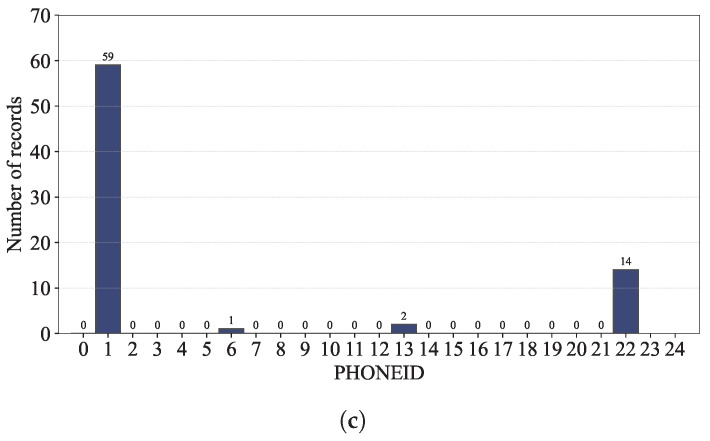
The number of WAP-absent records per (**a**) building, (**b**) user and (**c**) phone of the training dataset.

Note that the left-most bar of the histogram of Figure 15 indicates that there are few records with few or no detected WAPs. As the records with no detected WAPs, namely WAP-absent records, can impair the performance of indoor localization by mapping the same fingerprint of RSSIs of non-detected WAPs (The RSSIs of non-detected WAPs of the UJIIndoorLoc database are typically set to −110 before training a localization model [13].) to multiple locations, it is imperative to investigate WAP-absent records. Figure 16 shows the numbers of WAP-absent records per building, user and phone of the training dataset.

From Figure 16a, we observe that Building 0 has significantly fewer WAP-absent records than Building 1 and 2, while Figure 16b,c show that USER 8 and 17 and PHONE 1 and 22 are mostly associated with WAP-absent records.

Note that the validation dataset does not contain WAP-absent records, which could be explained as follows:

- PHONE 1 and 22 were not used for the validation dataset. The WAP-absent records, therefore, could be related with the sensitivity of specific phones as shown in Figure 16.
- The number of records of the validation dataset is significantly lower than that of the training dataset, so there were fewer chances of collecting WAP-absent records during the construction of the validation dataset.
- As the records of the validation dataset were collected three months later than those of the training dataset, the environment had changed and/or some WAPs had been removed or gone down.

We also investigate how many WAPs are detected at more than one building, which could impair the performance of indoor localization as well. The results are summarized in Table 4, where $L_{i}$ denotes the number of WAPs detected at Building *i* for i∈{0,1,2} and their combinations.

We can see that the number of WAPs detected at Building 1 and 2 is higher than that at Building 0 and 1, which is understandable given the shorter distance between Building 1 and 2 as shown in Figure 5a. The existence of WAPs detected at Building 0 and 2 and all three buildings, however, is unexpected, and their effects on localization and related pre-processing would be interesting topics for further investigation.

**Figure 17:**
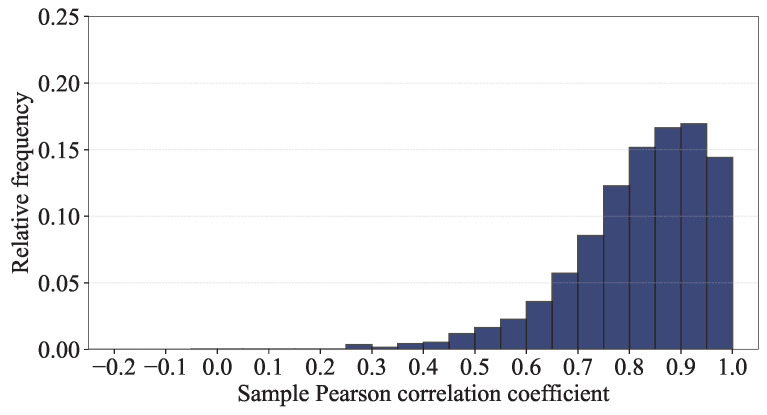
Histogram of the sample PCCs for all possible pairs of the RSSI samples measured at the same RPs in the training dataset.

In addition to the building-level analyses, we carry out an RP-level analysis of the RSSI samples measured at the same RPs based on the sample Pearson correlation coefficient (PCC), which measures the linear correlation between two samples [28,29]. For each pair of the RSSI samples of $X=[x_{1},\ldots ,x_{520}]$ and $Y=[y_{1},\ldots ,y_{520}]$ measured at an RP, their sample PCC is given by

(1) $$r_{X,Y}=\frac{\sum_{i=1}^{520}\left(x_{i}-\bar{x}\right)\left(y_{i}-\bar{y}\right)}{\sqrt{\sum_{i=1}^{520}{\left(x_{i}-\bar{x}\right)}^{2}}\sqrt{\sum_{i=1}^{520}{\left(y_{i}-\bar{y}\right)}^{2}}},$$

where

(2) $$\bar{x}=\sum_{i=1}^{520}x_{i}\;\mathrm{and}\;\bar{y}=\sum_{i=1}^{520}y_{i},$$

and $r_{X,Y}$ ranges from −1 to 1, where 1 indicates a positive correlation, 0 indicates no correlation and −1 indicates a negative correlation. Figure 17 shows the histogram of the sample PCCs for all possible pairs of the RSSI samples measured at the same RPs in the training dataset, which indicates strong positive correlations for most pairs.

There are still a few pairs with very low correlations; samples common to those pairs could impair the localization performance of a model and, therefore, should be filtered out as outliers.

### 3.4. Preliminary Experimental Results

To demonstrate potential benefits of the analyses presented in Section 3.1, Section 3.2 and Section 3.3, we carry out preliminary experiments based on them. All the experiments were run on a workstation with an Intel Core i9-9900X CPU, 128 GB RAM and two Nvidia GeForce RTX 2080Ti GPUs running Ubuntu 20.04.2 LTS, and all the models are implemented using Python 3.8.5. As the metric of the indoor localization performance, we use the EvAAL 3D error [19].

First, we assess the effects of WAP-absent records on indoor localization performance using the hierarchical RNN [17], SIMO DNN [13] and simple DNN and CNN models [30]. For the simple DNN and CNN models, we use the original architectures with a slight modification of their output nodes from 4 to 10 and their loss functions for multi-label classification and regression. As discussed in Section 3.3, the results summarized in Table 5 clearly indicate that we can improve the 3D error of all the models considered by filtering out the WAP-absent records from the database. Note that, though the building-floor hit rates of the SIMO DNN and simple DNN and CNN models slightly decrease without WAP-absent records, the building-floor hit rate is already taken into account in the calculation of the 3D error [19].

Second, we consider more complicated pre-filtering to exclude WAPs not providing unique information; specifically, we filter out (1) WAPs with less than 3 unique RSSI values and (2) WAPs with RSSI standard deviations less than 1.

After we apply the proposed pre-filtering to the training dataset, we also exclude in the validation dataset the WAPs that are already pre-filtered from the training dataset. As a result, the total number of WAPs of both training and validation datasets is reduced from 520 to 416, which also significantly reduces the training dataset size from 51,518 KB to 33,640 KB.

**Figure 18:**
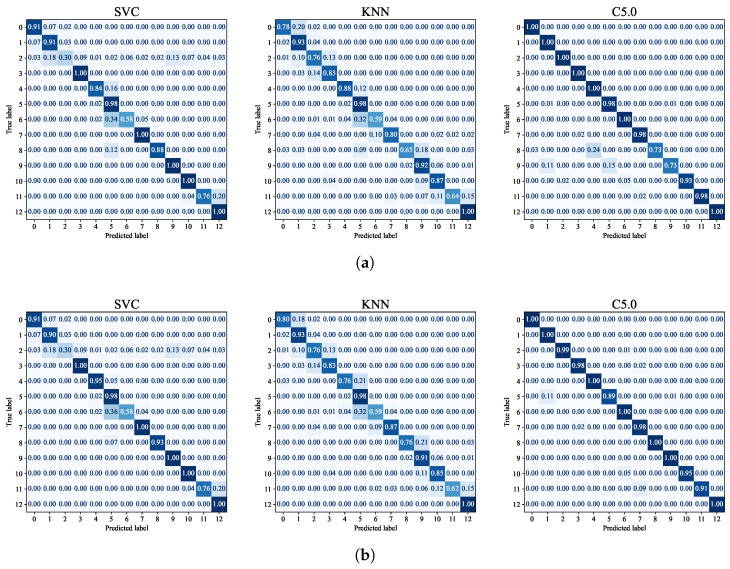
Confusion matrices of the building-floor classification based on SVC, KNN and C5.0: (**a**) without WAP-wise feature filtering and (**b**) with WAP-wise feature filtering.

To investigate the effects of the pre-filtering process, we evaluate the performance of building-floor classification using the conventional ML algorithms of KNN [31], C-support vector classification (SVC) [32] and C5.0 decision tree algorithm [33] as in [34]. Figure 18 shows the confusion matrices with and without pre-filtering, whose label mapping is given in Table 6.

From the results of Figure 18, we can observe that the filtering improves the classification performance slightly over all the algorithms. Note that, however, the major benefit of the filtering is the significant reduction of training time from 5 min to 3 min, i.e., by 40%.

We also evaluate the 3D errors as well as the building-floor hit rates of the hierarchical RNN, SIMO DNN and simple DNN and CNN models. The results are summarized in Table 7, which demonstrate that the pre-filtering improves both 3D errors and building-floor hit rates of all the models considered except the simple DNN model.

Through the preliminary experiments using different types of conventional ML and deep learning models with different metrics, we demonstrate that the proposed pre-filtering process based on the analyses of the UJIIndoorLoc database can improve the performance of indoor localization models in terms of accuracy, training time and generalization.

Finally, we investigate the effects of the representation of missing RSSIs (i.e., NaN) in the database by evaluating the localization performance with different numerical values. Table 8 summarizes the performance of the building-floor classification based on the conventional ML algorithms, and Table 9 provides the 3D errors and building-floor hit rates of the deep learning models [13,17,30].

The three numerical values for missing RSSIs are selected based on the following rationales:

- 100 is the original representation used in the UJIIndoorLoc database.
−105 is consistent with the minimal value of RSSIs, i.e., −104, in the UJIIndoorLoc database.
−110 is the most frequently used in the literature for research based on the UJIIndoorLoc database.

The results from Table 8 and Table 9 indicate that there is room for improvement in numerical representation of missing RSSIs.

## 4. Challenges and Recommendations

Section 3 reveals the issues of the fingerprints in the UJIIndoorLoc database in their spatial coverage, measurement practices and lack of diversity in users and phones, which could have negative effects on the performance of a localization model trained with the database. Here we provide our recommendations on the challenges in using existing fingerprint databases and constructing new ones for large-scale multi-building and multi-floor indoor localization.

### 4.1. On the Use of Existing Fingerprint Databases

#### 4.1.1. Localization Algorithms and Models

The accuracy of localization has been a top priority in studying localization algorithms and models, often at the expense of their space and time complexity, because the underlying scenario is that both training of a model during the offline phase and using a trained model for location estimation during the online phase are done at a centralized server with plenty of computing and power resources. Under this scenario, a user device just reports the RSSIs measured at an unknown location to and receives estimated location information from the server.

Achieving good localization accuracy is relatively straightforward with a fingerprint database constructed for a controlled small-scale environment over a short period of time, which, however, is challenging with a fingerprint database constructed for a large-scale multi-building and multi-floor environment over a long period of time. In the case of the UJIIndoorLoc database, the validation dataset was collected three months after the training dataset with fewer users and devices, resulting in fewer detected WAPs. To address the issues resulting from the differences between training and validation/testing datasets in large-scale multi-building and multi-floor indoor localization, techniques like transfer learning [35] and domain adaptation [36] could be applied for the improvement of the generalization and robustness of a model.

In addition to the accuracy and robustness of localization, user privacy is also an important factor. The conventional scenario based on the client-server model raises privacy concerns due to the collection of user information (i.e., location fingerprints) by a centralized server, especially during the online phase. As the computational power of user devices increase, it is possible to perform localization tasks directly on user devices (e.g., based on pretrained models downloaded from a server) without submitting user information to a server, which can protect users’ privacy.

As for response time, smaller models have an advantage over larger ones, which can also provide reasonable localization performance in small-scale indoor localization, but their direct application to large-scale indoor localization cannot guarantee the same level of performance. One promising solution in this regard is knowledge distillation [37], which can compress large models without significantly sacrificing their performance. This process has garnered much success in many research areas, but not yet in indoor localization.

Therefore, it is essential to strike the right balance between accuracy, robustness, response time and privacy in studying localization algorithms and models by taking into account the following important aspects:

- The number of records required for model training.
- The model size and computational power required.
- The amount of data collected and stored on the device.
- The structure and architecture of an indoor localization system (e.g., cloud-based and on-device).

#### 4.1.2. Data Balancing

The spatial complexity of building structures and the accessibility of RPs pose challenges to large-scale indoor localization, which lead to uneven spatial distribution of records. As discussed in Section 3.1, the UJIIndoorLoc database shows significant imbalance in the number of records (e.g., between the west and east corridors of Building 2). The UJIIndoorLoc database also shows the dominance of a couple of users and phones during the construction of both datasets. Such data imbalance in space, user and device distributions could result in poor and biased training results, so data balancing during the construction of the Wi-Fi fingerprint databases is essential to achieving unbiased results as well as good localization performance from trained models.

To handle data imbalance, we can apply data-augmentation and/or data-resampling techniques to existing fingerprint databases: as for data augmentation, straightforward application of conventional techniques (e.g., [25,38]) could provide satisfactory results when the original records already have a good space coverage like those of Building 0 shown in Figure 5b. When a building structure is complicated and original records poorly cover the space like the top floor of Building 2 or the bottom two floors of Building 1 of the UJIIndoorLoc database, however, we need a more sophisticated data-augmentation schemes like generative adversarial network (GAN)-based ones [39].

As for data resampling, we can apply advanced data-resampling techniques like stratified sampling [40] and weighted random sampling [41] as well as conventional up- and downsampling to obtain more evenly distributed datasets. For example, we can apply weighted random sampling to Floor 1 and 2 of Building 2 of the UJIIndoorLoc database.

#### 4.1.3. Data Preprocessing

Based on the comprehensive analyses in Section 3.2, it has been observed that certain WAPs are not playing a critical role in accurately identifying a specific building or floor. The significance of each WAP can vary depending on the particular building and floor. For instance, during data augmentation tasks, certain WAPs within the range of [20, 40] in the UJIIndoorLoc training dataset are detected in Building 0 but have negligible relevance for Building 2 as shown in Figure 13. Therefore, when selecting inputs for indoor localization models and complex data augmentation algorithms, it is crucial to consider the importance of each WAP at different levels.

It is also worth investigating the pre-filtering of WAPs with all-NaN values in the database. In the case of the UJIIndoorLoc database, as discussed in Section 3.2, Out of 520 WAPs, 55 and 153 WAPs were never detected in the training and validation datasets, respectively. Excluding these WAPs could substantially reduce the input dimension of indoor localization models and storage requirements for the database, particularly for large-scale scenarios. There have been proposed several WAP selection schemes [42,43,44,45], but their application to large-scale multi-building and multi-floor indoor localization databases are to be carefully investigated.

WAP-absent records in the database, i.e., resulting from the lack of WAPs around the user or the device’s inability to detect WAPs at the time of RSSI measurement, can deteriorate indoor localization performance and cause issues like the cold start problem [46], a decrease in robustness and poor generalization. In the case of the UJIIndoorLoc database, users with PHONE 1 or 22 are more likely to generate WAP-absent records on the top floor of Building 1 as discussed in Section 3.3. To avoid such issues, we can simply filter out WAP-absent records before training a model. We can also apply more advanced techniques like data imputation to handle such missing data [47].

#### 4.1.4. Missing Value Representation

It is important to understand that different databases may represent NaN with different numerical values. For example, the UJIIndoorLoc database labels NaN values as 100, which is typically converted to −110 during the preprocessing. While a larger variation in RSSI values for different locations could help room or floor classifications, it is unsuitable for coordinate regression as demonstrated in Table 8 and Table 9 of Section 3.4. Therefore, it is important to numerically represent missing RSSIs based on the statistics of RSSI values in the database, which can ensure accurate indoor localization for both classification and regression.

### 4.2. On the Construction of New Fingerprint Databases

Based on the case study in Section 3 and the discussions on the use of existing fingerprint databases in Section 4.1, we recommend the following guidelines for the construction of new fingerprint databases:

- RP selection should be carefully planned to cover monitoring areas in a balanced way.
- Given the limitation of labor resources, the frequency of RSSI measurements and the total period of database construction should be carefully determined.
- The diversity of users and devices should be considered during the construction of a fingerprint database.
- The strategy of periodical maintaining and updating the database should be deliberately designed.

The details of the guidelines are discussed in the following subsections.

#### 4.2.1. RP Selection

It would be ideal if we could tackle the issue of the uneven numbers of records per building, floor, user and phone during the construction of a database through a more systematic way of RP selection, because imbalanced data may cause issues like bias and overfitting in classification [48]. Proper selection of RPs during the construction of a database, however, is challenging in that the following, seemingly contradicting requirements should be met simultaneously:

- RPs should cover monitoring areas in a balanced way.
- RPs should be easily accessible for data collection.

As discussed in Section 3.1.1, the UJIIndoorLoc database shows the uneven numbers of records per building and floor due to the accessibility of the restricted spaces like chemical laboratories and private offices [18].

We can apply post-construction techniques as practical alternatives in this regard: for example, we can utilize spatial data augmentation [25] for Building 0 and 1 and stratified sampling [49] for Building 2. As a smaller number of RPs with balanced space coverage is better than a larger number of RPs with poor space coverage even for the application of the post-construction techniques, the selection of RPs was and remains a top priority for the construction of fingerprint databases.

#### 4.2.2. Measurement Practices

How to measure fingerprints based on the selected RPs is another important factor for not only the construction of a fingerprint database but also its maintenance and update.

The frequency of RSSI measurements (e.g., daily or weekly), together with the total period of database construction (e.g., over a month or a year), needs to be carefully determined. With a low measurement frequency, some WAPs may not be detected during the measurements due to their being in a standby or sleeping operation mode, while a high measurement frequency incurs a higher labor cost. Note that the effects of a low frequency of RSSI measurements could be compensated for using a longer period of database construction. As environment changes like people’s presence and the use of various electronic devices significantly affect the measured RSSIs, using different times of the day for measurements (e.g., during or after work hours) is also required to increase the temporal diversity of a fingerprint database.

Together with the measurement frequency and time and the database construction period, the way of visiting RPs is important, too. For example, we can visit and measure RSSIs at the same RPs repeatedly over a construction period for small-scale indoor localization, while we can divide RPs and visit a part of them during each measurement for large-scale multi-building and multi-floor indoor localization.

Though we carefully plan RSSI measurements at properly selected RPs for the construction of a fingerprint database, it will be useless if we do not have enough human resource to carry out the plan. As discussed in Section 2.6, a hybrid data-collection strategy combining in/outsourcing with volunteering can be adopted to reduce the high labor cost. Even with such a hybrid data-collection strategy, a core group of participants is still a key to the successful construction of a good fingerprint database, who is to provide high-quality RSSI measurements by strictly following the measurement plan.

#### 4.2.3. User, Device and Network Diversity

To increase the diversity of collected fingerprint data for the robustness of a trained model, the measurements should be done with multiple users of different physical characteristics (e.g., height) with different models of devices. For example, depending on a couple of devices could result in lots of WAP-absent records due to the special characteristics or even errors of certain devices as discussed in Section 3.3.

To further increase the diversity of location fingerprints and thereby provide more robust localization service in various indoor environments, we can collect and provide other types of location fingerprints like RSSIs of BLE [50] and cellular networks [51] and geomagnetic field intensity [52] as well as Wi-Fi RSSIs.

#### 4.2.4. Database Maintenance and Update

Once an indoor localization system is deployed in the field with a constructed fingerprint database, there will be increasing requirements for the maintenance and update of the fingerprint databases, the activities of which include addition of fingerprints from new WAPs, replacement of fingerprints from existing WAPs and removal of fingerprints no longer relevant.

In addition to manual collection of fingerprints by human participants, automatic collection of fingerprints using lightweight, battery-powered anchor devices deployed at selected RPs could be considered to further reduce the labor cost [53]. Also, exploiting unlabeled RSSIs submitted by users during the online phase of an indoor localization system deployed in the field is another interesting option in this regard [54].

## 5. Conclusions

Wi-Fi fingerprinting has become a dominant technology for indoor localization due to its major advantage of usability in any environment equipped with Wi-Fi networks without requiring additional hardware or infrastructure. As the localization performance of Wi-Fi fingerprinting heavily depends on the quality of the underlying fingerprint database used to train an ML model for location estimation, a study on the use and construction of fingerprint databases becomes as important as that on localization algorithms and models, whose major focus, however, has been limited to databases covering a single floor or building.

The UJIIndoorLoc Wi-Fi fingerprint database represents a significant advancement in the field of multi-building and multi-floor indoor localization. While many researchers have used this database as a benchmark to evaluate the performance of their proposed algorithms and models, there have been few studies dedicated to multi-building and multi-floor fingerprint databases. This paper aims to fill this gap by examining the UJIIndoorLoc database, which is by far the most popular multi-building and multi-floor Wi-Fi fingerprint database, and providing practical guidance on the use of existing databases and future directions for the design and construction of new databases.

As a basis, we have carried out a comprehensive case study of the UJIIndoorLoc database, where we investigate the statistical characteristics of the UJIIndoorLoc database through both field-level and record-level analyses. We have obtained several key insights on the UJIIndoorLoc database through those analyses, i.e., (1) the uneven spatial distributions of records, (2) the lack of user and phone diversity during the measurements, (3) the existence of WAP-absent records and (4) the identification of WAPs not providing unique information for fingerprinting; especially, we have assessed and demonstrated the effects of WAP-absent records and WAPs not providing unique information on indoor localization performance through preliminary experiments.

Based on the results of the case study with the UJIIndoorLoc database, we have provided our recommendations on the challenges in using existing fingerprint databases and constructing new ones for large-scale multi-building and multi-floor indoor localization, where we discuss in detail potential application of advanced ML techniques like data augmentation, data resampling, data imputation and semi-supervised learning in addressing the challenges.

To the best of the authors’ knowledge, this is the first work to extensively analyze the UJIIndoorLoc database, identify its issues and provide recommendations on the various challenges in using the existing database and constructing new ones for large-scale multi-building and multi-floor indoor localization. Our findings presented in this paper serve as a valuable starting point to researchers new to this field and provide a practical guidance to those interested in using the UJIIndoorLoc database or creating their own large-scale Wi-Fi fingerprint databases. Note that we have been constructing our own large-scale multi-building and multi-floor Wi-Fi fingerprint database based on the results of this case study, whose preliminary results are presented in [53].

## Figures and Tables

**Table 1:** A summary of publicly available Wi-Fi RSSI fingerprint databases.

Category	Implementation	[18]	[20]	[21]	[22]	[23]	[24]
Coverage	Single-floor		✓				
	Single-building and multi-floor			✓	✓	✓	
	Multi-building and multi-floor	✓					✓
Collection	Crowdsourcing		✓	✓			
	Insourcing	✓			✓	✓	✓

**Table 2:** Fields of the UJIIndoorLoc fingerprint label.

Field	Description	Range of Values
BUILDINGID	Building IDs	[0, 2]
FLOOR	Floor IDs	[0, 4]
LONGITUDE	Longitude	[−7695.9, −7300.8] *
LATITUDE	Latitude	[4,864,745.7, 4,865,017.4] *
SPACEID	Room IDs	[1, 254]
RELATIVEPOSITION	Room/corridor	[1, 2]
USERID	User IDs	[0, 18]
PHONEID	Phone IDs	[0, 24]
TIMESTAMP	Capture time	- **

* One decimal point only. ** No range is given due to the three-month interval.

**Table 3:** Building-level RP distribution and the average number of records per RP of the training dataset.

BUILDINGID	Number of RPs	Number of Records	Average Number of Records per RP
0	259	5249	20.3
1	265	5196	19.6
2	409	9492	23.2

**Table 4:** The number of WAPs detected at building(s).

Dataset	Building	Number of Detected WAPs		
Training	0	$L_{0}=200$	$L_{0,1}=59$	$L_{0,1,2}=3$
	1	$L_{1}=207$	$L_{1,2}=82$	
	2	$L_{2}=203$	$L_{0,2}=7$	
Validation	0	$L_{0}=183$	$L_{0,1}=46$	$L_{0,1,2}=2$
	1	$L_{1}=170$	$L_{1,2}=65$	
	2	$L_{2}=125$	$L_{0,2}=3$	

**Table 5:** Localization performance of the deep learning models with and without WAP-absent records.

Model	WAP-Absent Records	3D Error [m]	Building-Floor Hit Rate [%]
RNN	Without	**8.54**	**91.6**
	With	9.21	90.1
SIMO DNN	Without	**9.37**	90.4
	With	9.88	**91.9**
Simple DNN	Without	**10.43**	87.2
	With	10.94	**88.8**
Simple CNN	Without	**10.78**	87.1
	With	11.10	**89.7**

**Table 6:** Label mapping for the building-floor classification experiments.

BUILDINGID	FLOOR	Label
0	0	0
	1	1
	2	2
	3	3
1	0	4
	1	5
	2	6
	3	7
2	0	8
	1	9
	2	10
	3	11
	4	12

**Table 7:** Localization performance of the deep learning models with and without WAP-wise feature filtering.

Model	Pre-Filtering	3D Error [m]	Building-Floor Hit Rate [%]
RNN	Without	9.21	90.1
	With	**8.51**	**92.0**
SIMO DNN	Without	9.88	91.9
	With	**9.49**	**92.7**
Simple DNN	Without	10.94	**88.8**
	With	**10.72**	88.1
Simple CNN	Without	11.10	89.7
	With	**9.51**	**90.5**

**Table 8:** Building-floor classification performance of SVC, KNN and C5.0 with different numerical values for missing RSSIs.

Representation	Building-Floor Hit Rate [%]		
	SVC	KNN	C5.0
100	48.7	67.2	94.7
−105	**63.6**	**81.7**	95.2
−110	59.4	81.1	**95.9**

**Table 9:** Localization performance of the deep learning models with different numerical values for missing RSSIs.

Model	Representation	3D Error [m]	Building-Floor Hit Rate [%]
RNN	100	11.88	80.4
	−105	**8.28**	**94.0**
	−110	9.21	90.1
SIMO DNN	100	12.46	79.2
	−105	**9.56**	91.0
	−110	9.88	**91.9**
Simple DNN	100	15.63	73.4
	−105	**10.56**	86.3
	−110	10.94	**88.8**
Simple CNN	100	15.24	71.4
	−105	**10.89**	**90.5**
	−110	11.10	89.7

## Data Availability

No new data were created or analyzed in this study. Data sharing is not applicable to this article.
